# A cohort study using IL-6/Stat3 activity and PD-1/PD-L1 expression to predict five-year survival for patients after gastric cancer resection

**DOI:** 10.1371/journal.pone.0277908

**Published:** 2022-12-01

**Authors:** Xiao Ning Li, Yun Hong Peng, Wen Yue, Lin Tao, Wen Jie Zhang

**Affiliations:** 1 Department of Pathology, the First Affiliated Hospital, Shihezi University School of Medicine, Shihezi, Xinjiang, China; 2 Key Laboratories for Xinjiang Endemic and Ethnic Diseases, Shihezi University School of Medicine, Shihezi, Xinjiang, China; 3 Department of Physical Examination, the First Affiliated Hospital, Shihezi University School of Medicine, Shihezi, Xinjiang, China; 4 Department of Pathology, the Affiliated Oncology Hospital, Fudan University School of Medicine, Shanghai, China; Islamic Azad University Damghan Branch, ISLAMIC REPUBLIC OF IRAN

## Abstract

**Objectives:**

The expression/activation of IL-6, p-Stat3, PD-1 and PD-L1 in gastric cancer (GC) tissues were examined to evaluate their abilities in predicting the survival prognosis in postoperative patients with GC.

**Methods:**

The clinicopathological data and paraffin-embedded tissues of 205 patients who underwent gastric cancer resection were collected at the First Affiliated Hospital of Shihezi University School of Medicine, and the patients were followed-up annually after surgery. Immunohistochemistry (IHC) was used to detect the expression of IL-6, p-Stat3, PD-1 and PD-L1 proteins using tissue microarrays derived from these patients. Statistical analyses were performed using non-parametric tests, Spearman’s correlation, ROC curves, Kaplan-Meier survival analysis, Cox single-factor and multifactor regression models. In comparison, the analyses were also performed for GC patients from public databases (407 patients from TCGA and 433 patients from GEO, respectively).

**Results:**

(1) The expression levels of IL-6, p-Stat3, PD-1 and PD-L1 in GC tissues were significantly higher than adjacent normal tissues (ANT) (81.01% vs. 52.78%, *P*<0.001; 100% vs. 93.41%, *P*<0.001; 58.58% vs. 40.12%, *P*<0.001; 38.20% vs. 26.90%, *P* = 0.025, respectively). The mean optical density (MOD) values of IL-6, p-Stat3, PD-1 and PD-L1 were significantly higher in GC tissues. (2) The higher the levels of IL-6 (*P*<0.001), p-Stat3 (*P*<0.001), and PD-L1 (*P* = 0.003) were, the worse the survival prognoses were observed, respectively, among GC patients. The expression of PD-1 was not correlated with the prognosis of GC patients (*P*>0.05). The lower the degree of cell differentiation (*P*<0.001) was, the worse the survival prognoses were observed among GC patients. (3) Independent risk factors for postoperative prognosis in GC patients included age (≥60 years old), poor cell differentiation, invasion depth (T3/T4), lymph node metastasis (N1-3), distant metastasis (M1), and high levels of IL-6 (2+/3+). (4) A multi-factor combination (cell differentiation+IL-6+p-Stat3+PD-1+PD-L1) appeared to be the best survival predictor for GC patients as indicated by AUC (AUC 0.782, 95% CI = 0.709, 0.856, *P*<0.001). This combination may be the optimal predictor for postoperative survival of GC patients. (5) The levels of IL-6, p-Stat3, PD-1 and PD-L1 correlated with the infiltration levels of various tumor-infiltrating immune cells. (6) The analyses of ROC curves, calibration, DCA and Kaplan-Meier (KM) survival curves in TCGA dataset confirmed that the nomogram model could accurately predict the prognosis in GC patients.

**Conclusions:**

(1) The expressed levels of IL-6, p-Stat3, PD-1 and PD-L1 are higher in GC tissues than in adjacent normal tissues. (2) The high levels of IL-6, p-Stat3 and PD-L1 are correlated with poor survival in GC patients. (3) The high levels of IL-6, p-Stat3, PD-1 and PD-L1 have influences in GC tumor microenvironment. (4) The multi-predictor combination of "IL-6+p-Stat3+PD-1+cell differentiation" serves as an optimal survival predictor for postoperative GC patients and better than the TNM staging system. As these molecules can be examined in preoperative biopsies, these observations may provide a useful guide for clinicians to strategize individualized surgical plans for GC patients before surgery.

## 1. Introduction

Gastric cancer (GC) is one of the common malignancies in the digestive system and poses a major threat to public health around the world. The GLOBOCAN 2020 data has shown that GC is the fifth most prevalent cancer and the third leading cause of cancer-related death worldwide. In China, the morbidity of GC ranks the second and the mortality of GC ranks the third in all malignant tumors [1].

Because early symptoms of GC are not typical, patients often have advanced diseases at clinical diagnoses. Furthermore, many GC patients are in advanced tumor-node-metastasis (TNM) stages when first diagnosed and often have poor prognosis after surgery. TNM staging system is well established for predicting prognostic for GC patients. However, TNM stages are not available before surgery and therefore, a method that can predict the prognosis of GC patients before surgery would be very helpful for physicians to determine surgical options as well as postoperative treatment decisions.

Interleukin-6 (IL-6), a multifaceted cytokine that mediates responses to infection, is involved in immune diseases and cancers [2]. The signal transducer and activator of transcription 3 (Stat3) can be activated by IL-6 through phosphorylation and plays a crucial role in carcinogenesis through tumor-associated immunosuppression [3]. STAT3 is involved in numerous biological processes including cell proliferation, survival, differentiation, and angiogenesis [4, 5]. Our previous studies have shown that IL-6/Stat3 signaling pathway and its downstream molecules, such as IL-17, are pro-inflammatory and play a role in colon cancer and breast cancer [6, 7].

PD-1 (CD279) is a key immune checkpoint receptor and is usually expressed by activated lymphocytes, including CD8+ T cells, CD4+ T cells, natural killer (NK) T cells, B cells, activated monocytes, and dendritic cells [8]. PD-1 is activated by its ligand, PD-L1 (CD274), to inhibit antigen-stimulated lymphocyte proliferation, migration and cellular production, ultimately leading to diminished effector T cell function and immune tolerance [9]. PD-L1 is expressed on T and B cells, macrophages, and dendritic cells and many studies have shown that the expression of PD-L1 in cancer cells is tightly regulated by multiple oncogenic signaling pathways, including JAK/STAT3 [10]. The PD-1/PD-L1 signaling pathway is closely associated with a variety of diseases including autoimmune diseases, malignancies, and infectious viral diseases [11].

Based on studies that IL-6/Stat3 and PD-1/PD-L1 signaling pathways play important roles in cancers [6, 7, 11, 12], of which these molecules obtainable in biopsies before surgery and, TNM staging is not available before surgery, we have tested the hypothesis that cancer-associated signaling molecules may serve as potential markers capable of predicting survival prognosis before surgery among followed-up GC patients.

## 2. Materials and methods

### 2.1 Patients and samples

GC patients (205) of ethnic Han nationality patients after radical GC gastrectomy from January 2008 to January 2016 were collected from the First Affiliated Hospital of Shihezi University School of Medicine. All patients had complete clinico-pathological statistics. There were 154 males and 51 females. The median follow-up time was 64 months (range, 32–86 months) (see S1 Table for basic clinical information of GC patients studied). Patient inclusion criteria were as follows: (1) none received pre-operative chemotherapy or radiation therapy; (2) all patients underwent radical GC gastrectomy for the first time; and (3) no cancer remained after surgery.

### 2.2 Follow-up

The follow-up included telephone call-back, outpatient interview, and home visiting. The follow-up ended on October 1, 2019. The median following-up time was 64 months (range, 32–86 months). Overall survival (OS) was defined from the date of surgery until the date of death or the date of the last follow-up. Patients who died within 30 days after surgery were defined as 0 month survival. Patients’ informed consent was obtained orally by phone during follow-up or interview communications.

### 2.3 Classifications of gastric cancer

TNM (tumor-node-metastasis) clinical staging (I-IV), depth of invasion (T1-T4), lymphatic metastasis (N0-N3) and distant metastases (M0-M1) were defined according to the American Joint Committee on Cancer (the 8th edition, 2017) [5]. Gastric adenocarcinoma was classified according to the histopathological classification criteria of the World Health Organization (WHO, 2019) as follows: highly, moderately, and poorly differentiated adenocarcinomas [6].

### 2.4 Histological review

All resected tumor specimens were fixed with formalin and then stained with hematoxylin and eosin (H&E). All H&E slides were carefully reviewed by two senior pathologists. Tumor adjacent normal tissues (ANT) were taken from the area beyond 5 cm of the tumor tissue margin.

### 2.5 Construction of tissue microarrays (TMA)

The slides were independently reviewed by two senior pathologists to select the most representative sections. The most representative tumor area was carefully marked on the H&E-stained slide of each sample tissue. A TMA was constructed using 2-mm-diameter cores derived from the representative tumor areas and corresponding adjacent to carcinoma areas selected at random of formalin-fixed paraffin-embedded (FFPE) tissue blocks from each case in the Immunohistochemistry Laboratory of our hospital. TMAs were sectioned at a thickness of 4-μm and transferred to glass slides.

### 2.6 Immunohistochemistry

Immunohistochemistry (IHC) was performed to detect expressed proteins of IL-6 (rabbit anti-human polyclonal antibody, 1:200 dilution, ab6672, Abcam, USA), p-Stat3 (rabbit anti-human monoclonal antibody, 1:100 dilution, ab76315, Abcam, USA), PD-1 (mouse anti-human monoclonal antibody, 1:50 dilution, ab52587, Abcam, USA) and PD-L1 (rabbit anti-human monoclonal antibody, 1:100 dilution, ab205921, Abcam, USA), according to manufacturer’s instructions. HRP-conjugated second antibody and DAB kit were used to visualize antibody binding. Immunostaining reactivity was observed under light microscopy.

### 2.7 Evaluation of immunohistochemical staining

IHC slides were scored independently by two senior pathologists. Expression of IL-6 and p-Stat3 proteins was evaluated by a semi-quantitative grading method. The intensity of immunostaining was graded as follows: 0 = absent; 1 = Pale brown-yellow; 2 = Brown-yellow; 3 = Dark brown. The percentage of positive cells (the number of positive cells stained in the same field as a percentage of the total number of cells) was scored according to the following criteria: 0 ≤ 5%; 1 = 6%-25%; 2 = 26%-50%; 3 = 51%-75%; and 4 = 76%-100%. The overall score was obtained by multiplying the staining intensity score with the percentage score. The total scores ranged from 0 to 16: negative (0/-) = 1 score, weak positive (1+) = 2–4 score, positive (2+) = 5–8 score, strong positive (3+) = 9-12score.

Expression of PD-1 protein was evaluated by cell counting method [12]. IHC slides were observed completely under low-power microscope, and five high power fields (×400) were randomly selected in areas with high lymphocyte density. Counting the number of PD-1 positive cells (tumor cells and lymphocytes) in each field, and calculating the average values as the number of PD1-positive cells in this case. The mean of the number of PD-1 positive cells in all cases was used as the threshold. Above this threshold was regarded as PD-1 positive and below this threshold was PD-1 negative. The expression of PD-L1 on the cell membrane was scored by tumor cells and tumor-infiltrating immune cells. Specimens with ≥5% membranous expression were considered "positive".

To further determine the expression levels of IL-6, p-Stat3, PD-1 and PD-L1, semi-quantitative analysis was conducted by calculating the mean optical density (MOD) using Image J v. 1.8.0 software (the National Institutes of Health, Bethesda, MD, USA). Briefly, photographs were collected at a magnification of X400 under the same exposure conditions. Five random photographs were captured for each slice. The MOD value of 1 slice was calculated as the mean value of 5 randomly selected fields on the slice.

### 2.8 Public data sources

Transcriptome data (FPKM value) and clinical information of GC patients were obtained from the TCGA database (407 cases) (https://portal.gdc.cancer.gov/) and from the GEO (GSE84437) database (433 cases) (https://www.ncbi.nlm.nih.gov/geo/). Transcriptome data (FPKM value) was transformed into TPM in the TCGA database by batch correction and homogenization using "limalma" and "sva" packages. GC samples in the TCGA and GSE84437 were combined and summarized according to gene consistency. All GC cases with complete gene expression data and clinical information were included in our analysis.

### 2.9 Bioinformatics analysis

In accordance with the median expression value, IL-6, Stat3, PD-1 and PD-L1 mRNA expression levels were divided into high level group and low level group. The survival package of R software was used for visualization, and the Kaplan-Meier survival curves were obtained. GSEA was used to explore the signaling pathways related to IL-6, Stat3, PD-1 and PD-L1 in GC. Gene expression enrichment analysis was carried out between datasets with low or high IL-6, Stat3, PD-1 and PD-L1 mRNA expression. The annotated gene set was selected (c2.cp.kegg.v7.4.symbols.gmt) as the reference gene set. The normalized enrichment score (NES), nominal p-value, and false discovery rate (FDR) q-value indicated the importance of the association between gene sets and pathways. The constituent ratios of 22 tumor-infiltrating immune cells in different samples were inferred by the de-convolution algorithm using the CIBERSORT analysis tool. The ESTIMATE method was used to evaluate the immune score, stromal score, and ESTIMATE score of each sample. *P* < 0.05 indicated reliable inferred cell composition. According to the risk score, samples were divided into high and low risk groups, and Kaplan-Meier (K-M) survival analysis was used to compare survival differences between the above two groups. ROC curves were performed to evaluate the accuracy of the nomogram in TCGA cohort. Calibration curves were constructed to determine whether the predicted survival was consistent with the actual survival. In addition, decision curve analysis (DCA) was conducted to assess the clinical outcomes of decision strategies.

### 2.10 Statistical analyses

The Kruskal-Wallis H test was used to compare differential expression of the four immunohistochemical indexes between gastric and paraneoplastic tissues and their correlation with the clinicopathological data of postoperative GC patients. Spearman rank correlation was used to analyze the correlation between the pathological data and immunohistochemical indexes. Univariate and multifactorial Cox regression risk models were used to analyze the influence of postoperative survival of gastric cancer patients. The Kaplan-Meier method was used to analyze the relationship between the degree of differentiation, TNM stage and the expression of four immunohistochemical indexes and the prognosis of gastric cancer patients after surgery. The logistic regression model combined with ROC curves was used to describe the predictive ability of the combined test on the survival status of patients after surgery. The survival and rms packages in R Studio software were used to create column line graphs. The Hosmer-Lemeshow goodness-of-fit test was used to analyze the agreement between the predicted values and the actual observed values of the combined multiplex test, and calibration curves were plotted. *P*<0.05 was considered statistically significant in our study. SPSS 22.0 software, the R (version 4.2.0) and R Bioconductor packages were used for all data analysis.

### 2.11 Ethics approval and consent to participate

Ethical approval was obtained from the Institutional Ethics Review Board (IERB) of the First Affiliated Hospital, School of Medicine, Shihezi University (No. 2018-067-01). The IERB waived the need for patient consents due to anonymous analyses of the data and confidentiality and anonymity in the handling and publication of patients’ tissues. Standard University Hospital Guidelines in accordance with the Declaration of Helsinki were followed in this study. The data from TCGA and GEO (GSE84437) are publicly available and exempted from the approval of local ethics committees. The current research followed the TCGA and GEO data access policies and publication guidelines.

## 3. Results

### 3.1 Expression of IL-6, p-Stat3, PD-1 and PD-L1 proteins in gastric cancer and adjacent tissues

**Fig 1:**
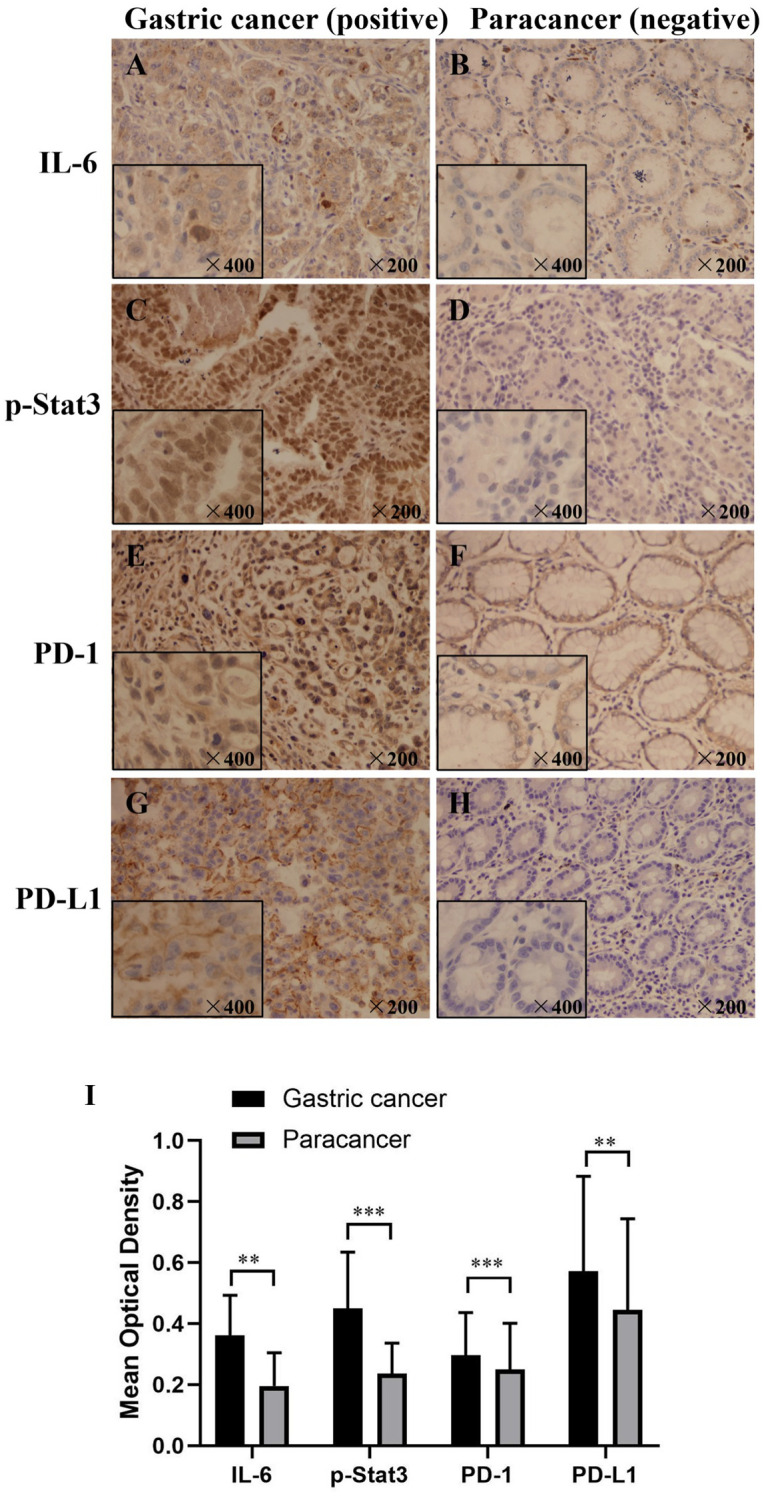
Expression of IL-6, P-Stat3, PD-1 and PD-L1 in GC and paracancerous tissues. (A), (C) and (G) were the positive expressions of IL-6, P-Stat3 and PD-L1 in gastric cancer tissues respectively(Envision ×200); (E) was positive expression of PD-1 in gastric cancer interstitial infiltrating lymphocytes(Envision ×200); (B), (D) and (H) were negative expressions of IL-6, P-Stat3 and PD-L1 in paracancer tissues respectively(Envision ×200); (F) is negative expression of PD-1 in gastric cancer interstitial infiltrating lymphocytes(Envision ×200); (I) Each image is partially enlarged at the bottom left (Envision ×400). The quantification is representative of the optical density in 5 fields ±SD by random. **P < 0.01,***P < 0.005.

The levels of IL-6, p-Stat3, PD-1 and PD-L1 proteins were determined by IHC and the MOD values were assessed. Fig 1 shows representative images of IL-6, p-Stat3, PD-1 and PD-L1 protein levels based on IHC, respectively. As shown in S2 Table, IL-6 positive staining was detected in 81.01% gastric cancers, and the positive rate was significantly higher in gastric cancer tissues than adjacent tissues (81.01% versus 52.78%, *P*<0.001). The expression of p-Stat3 protein was also higher in gastric cancer tissues than adjacent tissues (100% vs 93.41%, *P*<0.001). PD-1 positive staining was detected in 99 of 169 gastric cancers (58.58%), which was significantly higher than in adjacent tissues (58.58% vs. 40.12%, *P*<0.001). The expression of PD-L1 was also higher in gastric cancer tissues than adjacent tissues (38.20% vs. 26.90%, *P* = 0.025). Similarly, the MOD values of IL-6, p-Stat3, PD-1 and PD-L1 were significantly higher in GC tissues compared with the controls (*P* < 0.01).

### 3.2 Correlations between the factors and Clinicopathological Characteristics in gastric carcinoma

The correlation of IL-6, p-Stat3, PD-1 and PD-L1 proteins expression and other clinicopathologic factors is summarized in S3 and S4 Tables and Table 1. No significant differences were found in the IL-6 protein expression level depending on age, gender, differentiation, T stage (invasion depth). A high IL-6 protein expression level was found to be correlated with N stage (Regional Lymph Nodes) (r = 0.161, *P* < 0.05), M (Metastasis) (r = 0.206, *P* < 0.01) and TNM staging(r = 0.234, *P*< 0.01).

**Table 1:** Cross correlation analyses of various indices in gastric cancer patients.

Index	Clinical index							ImmunohistochemistryIndex			
	Gender	Age	Differentiation	T	N	M	TNM	IL-6	p-Stat3	PD-1	PD-L1
Gender	1										
Age	-0.072	1									
Differentiation	0.023	-0.069	1								
T	-0.019	0.098	**0.208** **	1							
N	-0.099	0.048	**0.190** **	**0.282** **	1						
M	0.067	0.098	0.057	0.071	**0.257** **	1					
TNM	-0.060	0.072	**0.234** **	**0.652** **	**0.712** **	**0.517** **	1				
IL-6	0.088	0.079	0.131	0.037	**0.161** *	**0.206** **	**0.234** **	1			
p-Stat3	-0.043	0.038	**0.158** *	**0.184** *	**0.313** **	0.118	**0.276** **	-0.066	1		
PD-1	0.044	0.029	0.083	0.092	-0.141	-0.057	-0.001	**0.243** **	0.024	1	
PD-L1	-0.102	0.050	**0.188** *	**0.202** **	0.135	0.114	**0.288** **	**0.234** **	**0.197** *	**0.176** *	1

Note:

* represents *P*<0.05,

** denotes *P*<0.01

The p-Stat3 protein expression in tumor tissue was associated with differentiation(r = 0.158, *P*<0.05), T stage(r = 0.184, *P*<0.05), N stage(r = 0.313, *P*<0.01) and TNM staging (r = 0.276, *P*<0.01). We did not found any significant association between p-Stat3 protein expression and other patients’ characteristics or clinicopathological characteristics (age, gender and M).

No statistical correlation was observed between clinicopathological factors and PD-1 protein expression(P > 0.05). PD-L1 protein expression was correlated with differentiation (r = 0.188, p < 0.01), T stage (r = 0.202, P = 0.008), and TNM staging (r = 0.288, P<0.001). Age, gender, N stage and M were not associated with PD-L1 protein expression.

We also found that IL-6 protein expression was positively correlated with PD-1 (r = 0.243, *P*<0.01), and PD-L1 (r = 0.234, *P*<0.01) protein expression. And PD-L1 protein expression was positively correlated with p-Stat3 (r = 0.197, *P*<0.05), and PD-1 (r = 0.176, *P*<0.05) protein expression.

### 3.3 Influence of differentiation, IL-6, p-Stat3, PD-1 and PD-L1 protein expression on the survival of GC patients after surgery

**Fig 2:**
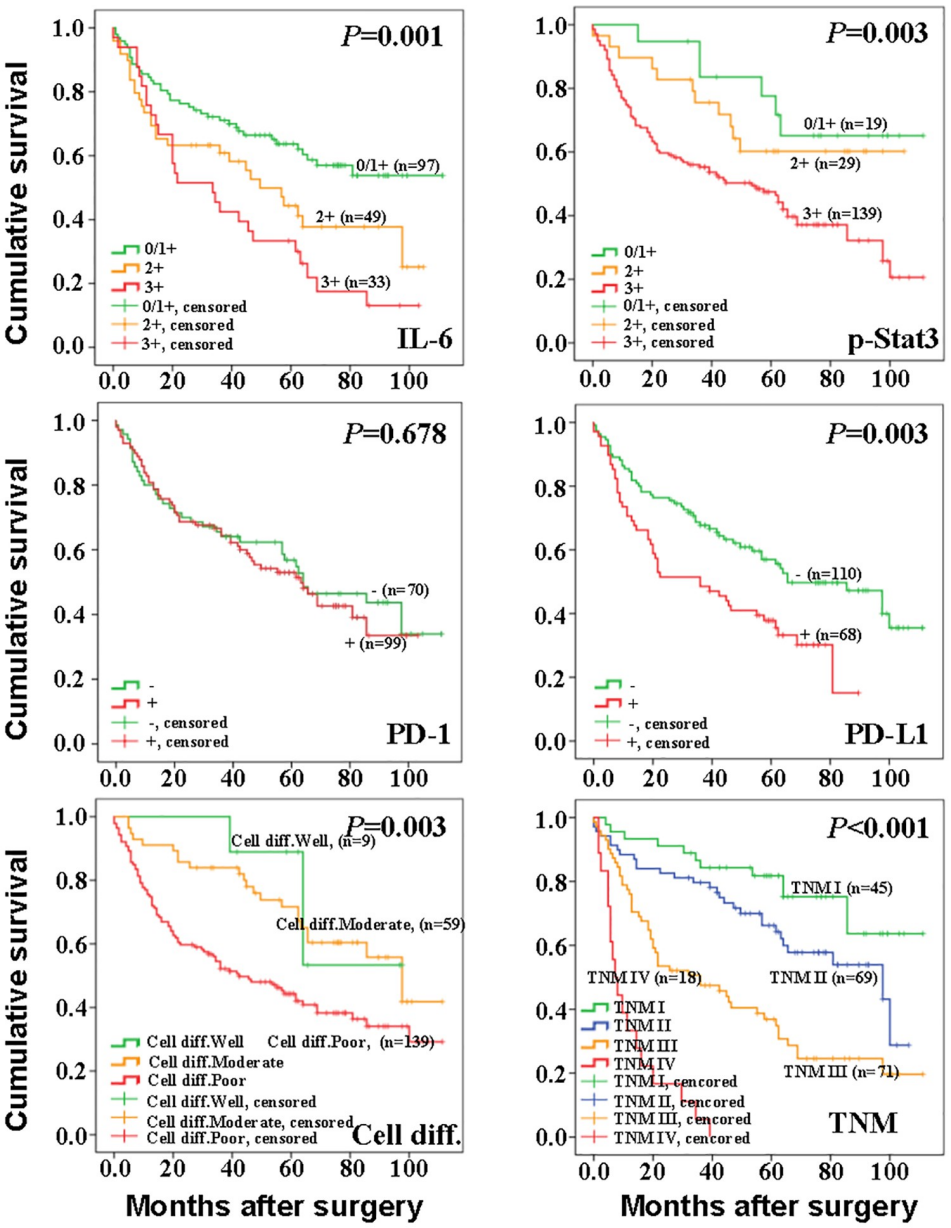
Effects of TNM stage, differentiation degree, IL-6, p-Stat3, PD-1 and PD-L1 on survival of gastric cancer patients. Impacts of TNM staging, cell differentiation, IL-6, p-Stat3, PD-1 and PD-L1 on the survival of GC patients. Comparisons of Kaplan-Meier survival curves for GC patients categorized as the following: 0/1+ vs. 2+ vs. 3+ of IL-6 (P = 0.001), p-Stat3 (P = 0.003),—vs. + of PD-1 (P = 0.678), PD- L1 (P = 0.003), respectively; clinical staging of TNM I vs. TNM II vs. TNM III vs. TNMIV (p<0.001); cell differentiations of well vs. moderate vs. poor (P = 0.003).

As shown in Fig 2, Kaplan-Meier survival curve indicted that cell differentiation (*P* = 0.003), TNM staging (*P* < 0.001), expression of IL-6(*P* < 0.001), p-Stat3(*P* = 0.003) and PD-L1(*P* = 0.003) protein significantly affected overall survival. The OS of patients was significantly reduced by poorly differentiated type cells, high expression of IL-6、p-Stat3 and PD-L1 protein, and TNM stage II~IV(*P* < 0.05). The expressions of PD-1 protein had no significant effect on the survival of patients.

### 3.4 Survival prediction power of different bio-indicators in GC patients after surgery

**Fig 3:**
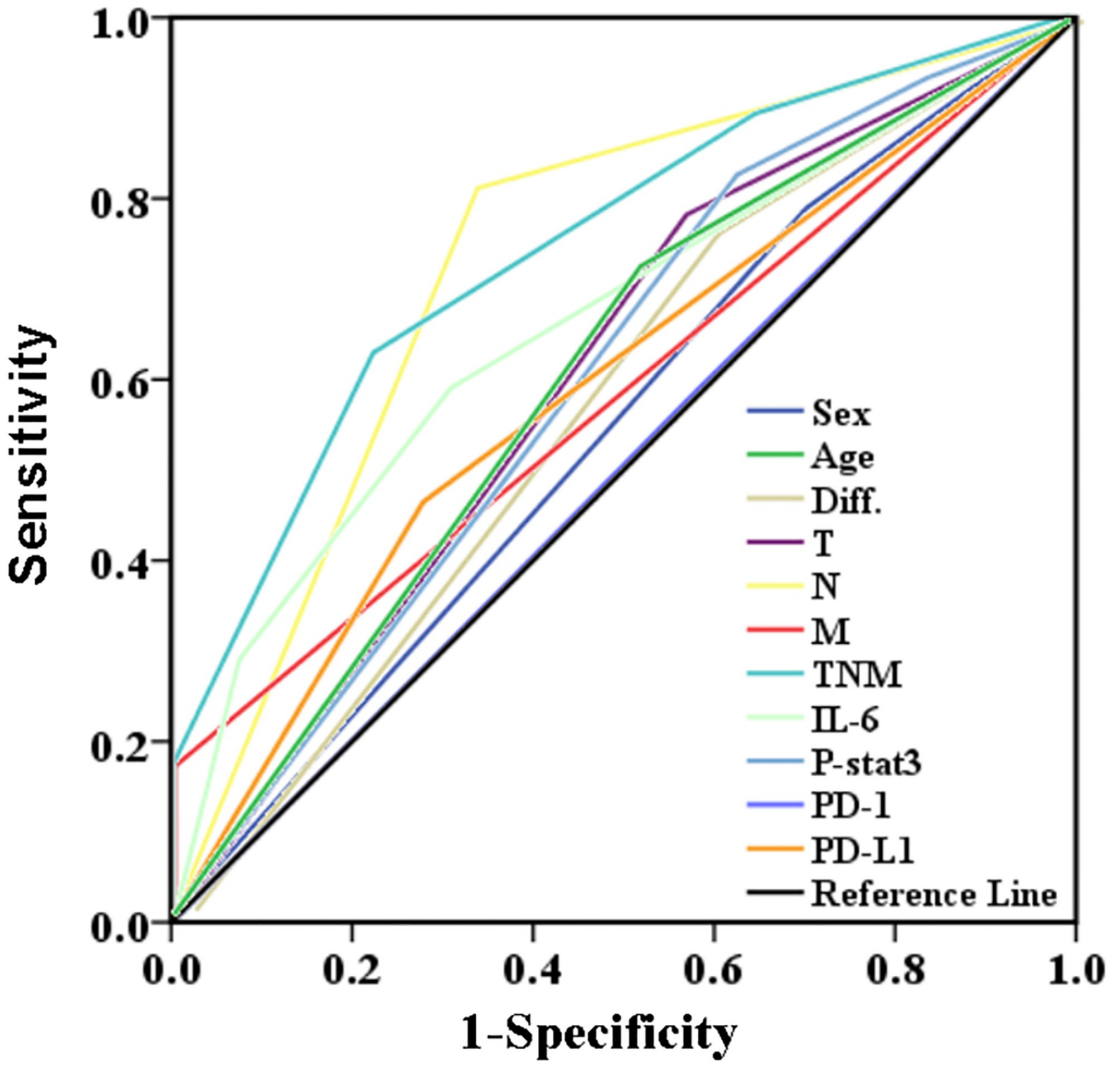
ROC curve of clinicopathological index and immunohistochemical index to predict survival of gastric patients. ROC curves reveal performance abilities of clinicopathological indices and IHC bio-indicators affecting patients’ survival. As shown, the diagonal black line is the reference line.

Survival prediction power is AUC-quantified probability of survival for a particular bio-indicator. The volume of AUC represents the weight of a potential factor in predicting survival prognosis. As shown in Fig 3, the ROC curves for the 11 potential factors showed differences, and their corresponding AUCs were displayed in Table 2. The ability of T, N, M and TNM staging to predict OS in GC patients has been recognized. In our study, the AUC of TNM staging was the largest. IL-6 protein expression had a comparable AUC (0.663) to well-established TNM staging (0.742). Differentiation(AUC = 0.588, *P* = 0.030), age (AUC = 0.592, *P* = 0.033), expression of p-Stat3 (AUC = 0.601, *P* = 0.017), and PD-L1 (AUC = 0.593, *P* = 0.033) protein also showed similar AUCs to those of TNM staging system although to a lesser degree.

**Table 2:** AUCs (area under the curves) for various bio-indicator in gastric cancer.

Index	AUC	95% CI	*P* values	Sensitivity	Specificity	Youden index
Gender	0.540	0.461–0.620	0.319	78.90%	29.17%	0.081
Age	0.602	0.524–0.680	**0.033**	72.48%	47.92%	0.204
Differentiation	0.588	0.509–0.667	**0.030**	76.15%	41.05%	0.172
T	0.607	0.529–0.686	**0.008**	78.90%	42.55%	0.215
N	0.738	0.667–0.809	**<0.001**	81.65%	65.96%	0.476
M	0.583	0.505–0.660	**0.043**	16.51%	100.00%	0.165
TNM stages	0.742	0.675–0.809	**<0.001**	62.39%	77.66%	0.401
IL-6	0.663	0.584–0.742	**<0.001**	58.95%	69.05%	0.280
p-Stat3	0.601	0.519–0.683	**0.017**	83.33%	36.47%	0.198
PD-1	0.503	0.416–0.591	0.941	58.89%	41.77%	0.006
PD-L1	0.593	0.509–0.677	**0.033**	46.46%	72.15%	0.186

Note: *P* <0.05 indicates significant statistical differences.

### 3.5 Risk factors affecting the prognosis of GC patients

The univariate Cox regression model revealed that age, differentiation, T, N, M, TNM staging, expression of IL-6, p-Stat3, and PD- L1 protein were associated with prognosis of GC patients in terms of OS (*P* < 0.05). In the multivariate analysis, age, differentiation, T, N, M, and IL-6 protein expression were independent prognostic factor for OS (*P* < 0.05). The factors of poorly differentiated type, age ≥60 years, TNM stage III/IV, T stage T3/T4, lymphatic, distant metastasis, high expression of IL-6, p-Stat3, and PD- L1 protein had significantly positive influences on the increased risk of death, with the hazard ratios (HRs) of 2.121, 1.728, 3.514, 2.494, 4.612, 6.695, 2.05, 2.666 and 1.836, respectively (Table 3).

**Table 3:** Cox regression model analyses of bio-indicators in gastric cancer.

Variable	COX univariate analysis		COX multivariate analysis	
	HR (95% CI)	*P* values	HR (95% CI)	*P* values
Gender(female vs male)	0.800(0.505–1.267)	0.341	/	/
Age(≥60years vs <60years)	1.728(1.135–2.632)	**0.011**	1.793(1.090–2.952)	**0.022**
Differentiation (low vs. high+moderate)	2.121(1.362–3.303)	**0.001**	1.928(1.132–3.286)	**0.016**
TNM stages (T₃+T4 vs. T1+T2)	2.494(1.568–3.967)	**<0.001**	2.073(1.053–4.080)	**0.035**
N(yes vs no)	4.612(2.833–7.509)	**<0.001**	3.495(1.709–7.146)	**0.001**
M(M_1_ vs M_0_)	6.695(3.880–11.550)	**<0.001**	3.534(1.804–6.923)	**<0.001**
TNM stages(III/IV vs I/II)	3.514(2.372–5.206)	**<0.001**	0.774(0.380–1.579)	0.482
IL-6(2+/3+ vs -/+)	2.050(1.360–3.090)	**0.001**	1.806(1.124–2.901)	**0.015**
p-Stat3(2+/3+ vs -/+)	2.666(1.166–6.094)	**0.020**	1.042(0.436–2.495)	0.926
PD-1(+/-)	1.093(0.716–1.669)	0.680	1.129(0.695–1.835)	0.624
PD-L1(+/-)	1.836(1.226–2.750)	**0.003**	1.793(1.090–2.952)	**0.022**

Note: *P* <0.05 indicates statistical differences.

### 3.6 Combination of multiple survival predictors to assess the prognosis of gastric cancer patients after surgery

Our study aims to enhance the ability to predict survival using combinations of multiple bio-indicators which can be obtained prior to surgery. We use the index reduction method to find models with fewer indicators and better predictive power. As mentioned above, ROC curves and AUC are means to quantify powers or abilities of survival predictors. As shown in S5 Table, the combination 2 (differentiation+IL-6+p-Stat3+PD-1) had an AUC of 0.782 (95% CI,0.709–0.856) (*P*<0.001), and had the strongest predictive power, similar to the full 5 molecular combination of differentiation + IL-6 + p-Stat3 + PD-1 + PD-L1, which had an AUC of 0.777 (95% CI,0.701–0.854) (*P*< 0.001). Surprisingly, the AUC of the differentiation+IL-6+p-Stat3+PD-1 combination was much higher than that of TNM staging, which is the accepted standard, that only produced an AUC of 0.742 (95% CI, 0.675–0.809) (*P* < 0.001). Therefore, the combination of differentiation+IL-6+p-Stat3+PD-1 would be the best combination to predict 5-year OS of GC patients after surgery.

### 3.7 Establishment and validation of a Nomogram for predicting 5-year OS

**Fig 4:**
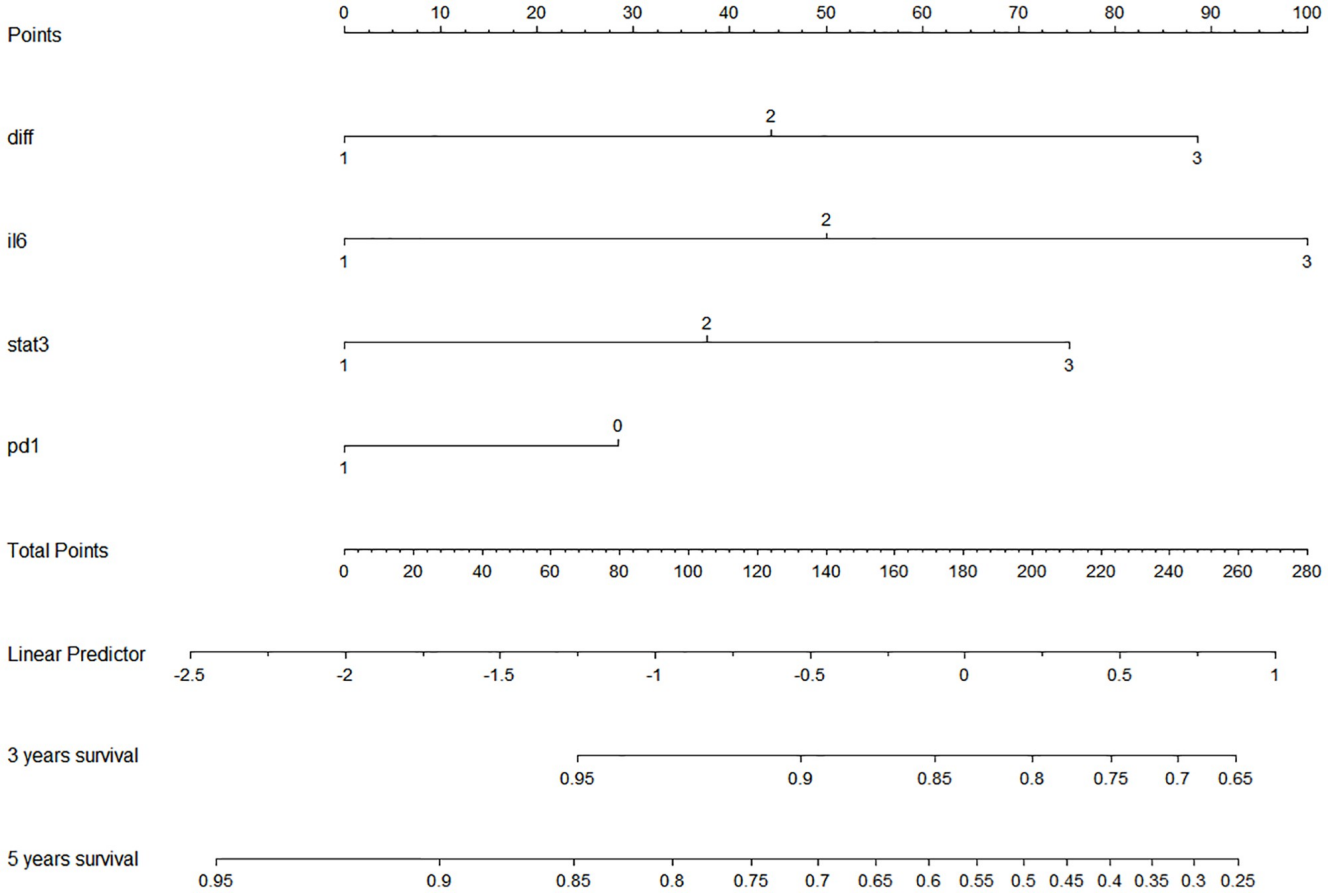
Column of postoperative survival of patients with gastric cancer predicted by differentiation +IL-6+ p-STAT3 +PD-1. The lengths of the differentiation, IL-6, p-Stat3, and PD-1 variables represent the contribution of that factor to the event of death in gastric cancer, upward corresponding to the scores labeled in Points. Depending on the individualized status of the patient, the scores for each variable are summed to obtain a Total Points value, and the scale is projected downward to yield a predicted survival value for that individual with gastric cancer at 3 or 5 years postoperatively.

To develop an intuitive and quantitative method to better stratify patients with different prognoses, a nomogram to predict 5-year OS was developed on the basis of the final model (Fig 4). Each variable was given a point according to HR. The probability of 5-year OS can be obtained by summing the total scores of each variable and locating them on the total score scale. The 5-year OS prediction accuracy was validated by the Hosmer-Lemeshow Goodness-of-fit (HLGOF) test which showed a correlation between the actual observed outcome and the prediction by the nomogram (χ^2^ = 7.963, *P* = 0.437) (S1 Fig).

**Fig 5:**
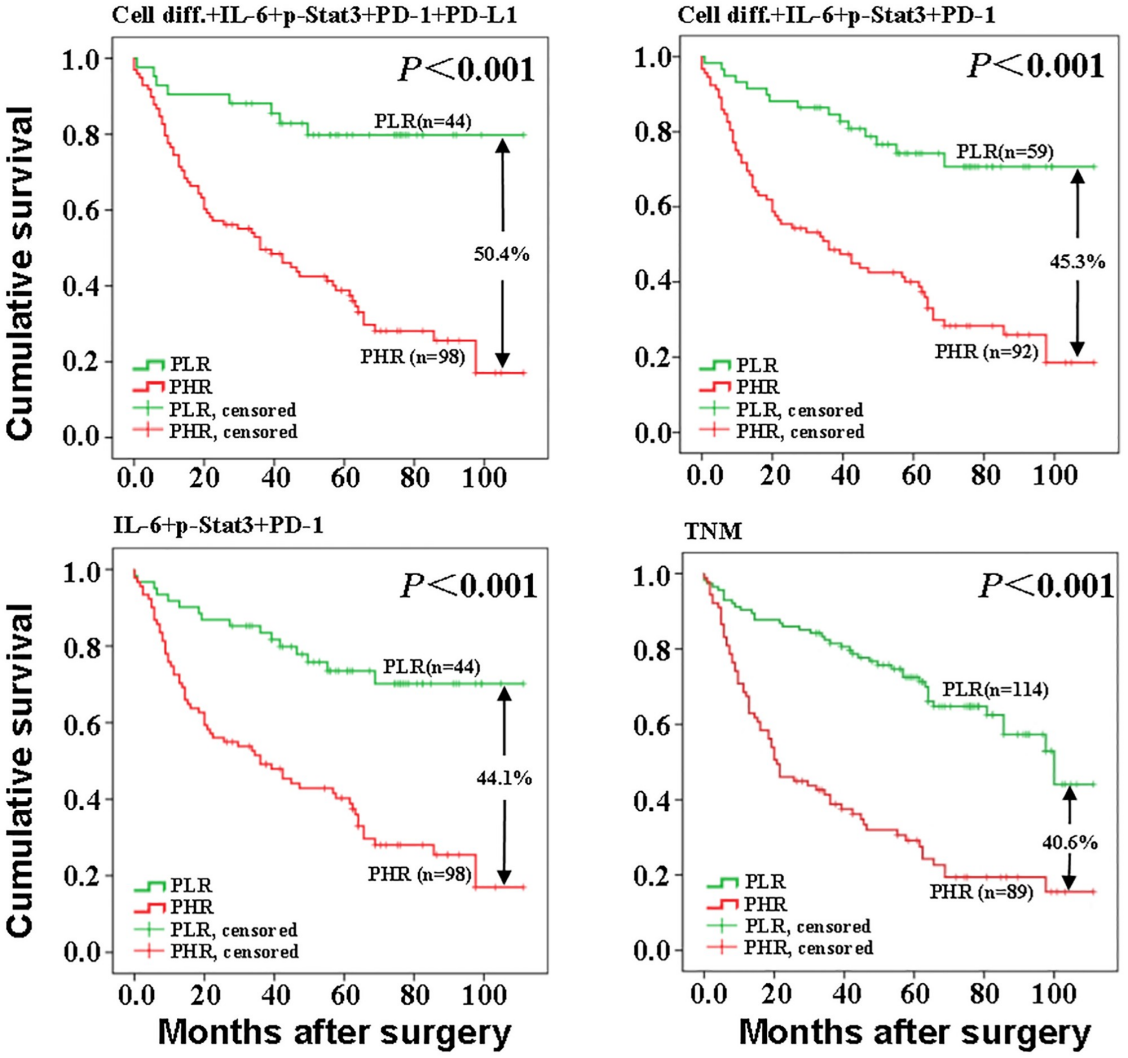
Prediction of low and high risk survival curves in patients with gastric cancer. The joint prediction model in this study was divided into a low mortality risk group (PLR) and a high mortality risk group (PHR), using the Cut-off value as the boundary. The cumulative survival rate of patients with gastric cancer in the low-risk group in combination 1 (81.0%) was higher than that of patients with gastric cancer in the high-risk group (30.6%), with a difference of 50.4%. In combination 2, the cumulative survival rate of gastric cancer patients in the low-risk group (74.6%) was higher than that of gastric cancer patients in the high-risk group (29.3%), with a difference of 45.3%. The cumulative survival rate of gastric cancer patients in the low-risk group (73.8%) in combination 3 was higher than that of gastric cancer patients in the high-risk group (29.7%), with a difference of 44.1%. The cumulative survival rate of gastric cancer patients in the low-risk group in combination 5 (TNM) (64.0%) was higher than that of gastric cancer patients in the high-risk group (23.6%), with a difference of 40.4%.

GC patients were grouped by the cut-off value (S5 Table) into predictive low risk (PLR) and predictive high risk (PHR). Kaplan-Meier survival curve indicted that three combinations had a wide discriminative distance between PLR and PHR groups and exhibited superior discriminative power to TNM staging. It also indicted that the reliability and rationality of differentiation+IL-6+p-Stat3+PD-1 as the optimal combination (Fig 5).

### 3.8 Expression levels of IL-6, Stat3, PD-1 and PD-L1 and their relationships with tumor immunity in combined TCGA and GEO gastric cancer datasets

**Fig 6:**
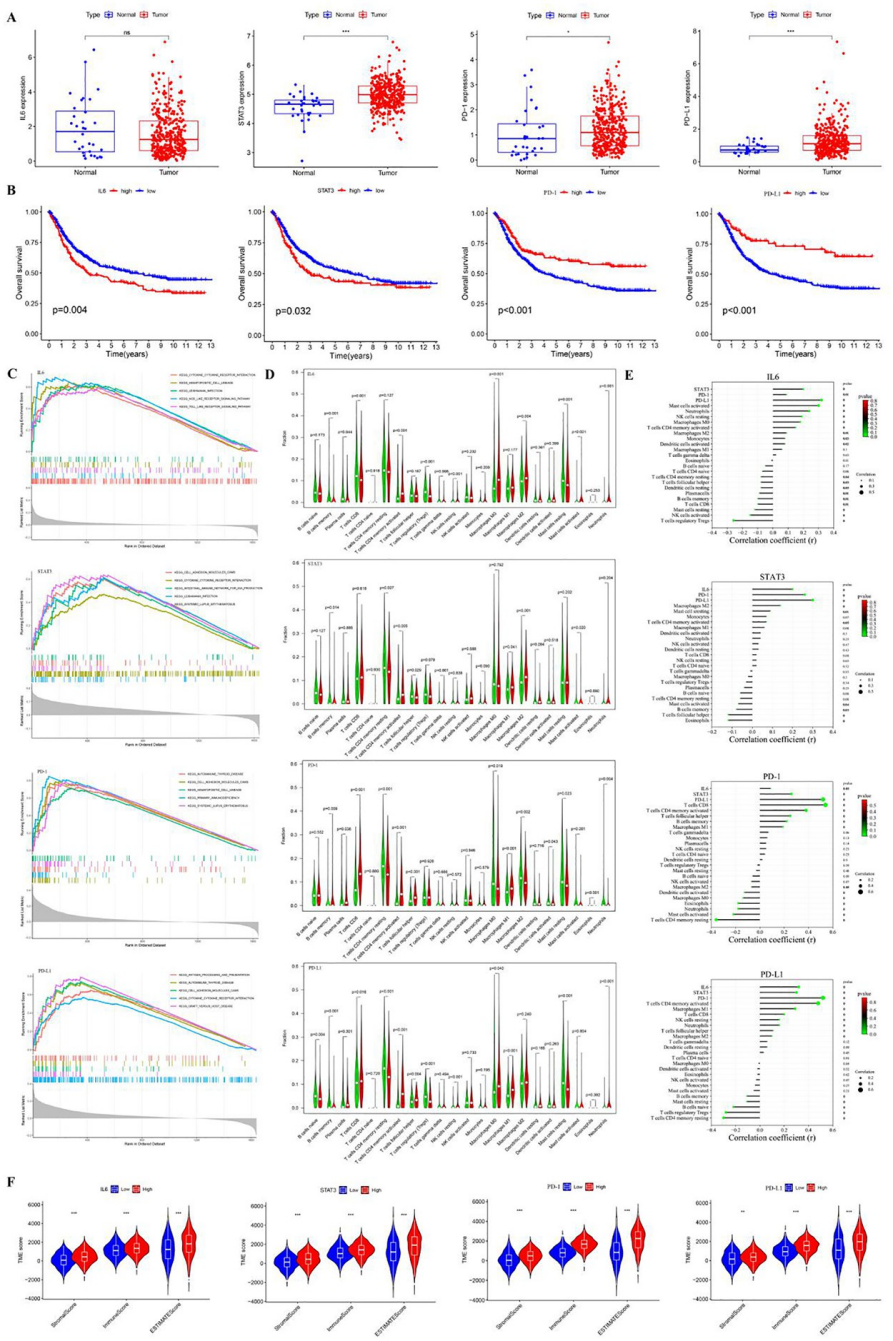
The expression of IL-6, Stat3, PD-1 and PD-L1 and their relationship with tumor immunity in TCGA and GEO combined gastric cancer datasets. The mRNA expression of STAT3/IL6/PD-1/PD-L1 and corresponding median of GC patients to classify the high-low rating group. (A) Expression levels of IL6, STAT3, PD-1 and PD-L1 between tumor and normal tissues of gastric cancer patients in TCGA database. (B) Kaplan-Meier curves for STAT3, IL6, PD-1 and PD-L1 expression in low- and high-risk groups of patients. (C) The merged enrichment plots of STAT3, IL6, PD-1 and PD-L1 from gene set enrichment analysis including enrichment score and gene sets. (D) Violin plots (Wilcoxlog-rank test) of the differences in 22 immune cell fractions between the high and low expression groups of STAT3, IL6, PD-1 and PD-L1. (E) The Correlation between immune checkpoint genes and immune components. (F) Correlation of STAT3, IL6, PD-1 and PD-L1 expression with TME (the immune score, stromal score, and ESTIMATE score).*P < 0.05, **P < 0.01, ***P < 0.001.

The expression levels of Stat3, PD-1 and PD-L1 differed in gastric cancer and normal tissues adjacent to the cancer in TCGA database. IL6 mRNA expression did not differ between cancerous and paracancerous tissues (Fig 6A).

IL-6 and Stat3 mRNA high expressing patients in the TCGA and GEO combined database have worse outcome (*P* < 0.05), consistent with our research results. PD-1 mRNA high expressing patients in the TCGA and GEO database also have better outcome (*P* < 0.001). PD1 also showed the same trend (*P* < 0.001) (Fig 6B).

On the basis of the TCGA and GEO combined data, we explored the function of IL-6, Stat3, PD-1 and PD-L1 and its related signal transduction pathway through GSEA. Combined with NES, FDR Q value, and nominal P value, 5 significantly enriched signaling pathways were selected. In this study, signaling pathways involved in cell adhesion molecules cams(CAMs), cytokine-cytokine receptor interaction, intestinal immune network for IgA production, leishmanin infection and systemic lupus erythematosus were differentially enriched in the highly expressed phenotypes of Stat3. The signaling pathways involved in cytokine-cytokine receptor interaction, nod like receptor signaling, Toll like receptor signaling and etc. were differentially enriched in the highly expressed phenotypes of IL-6. The cell adhesion molecules cams, hematopoietic cell lineage, primary immunodeficiency and etc. were differentially enriched in the highly expressed phenotypes of PD-1. The antigen processing and presentation, cell adhesion molecules cams, cytokine-cytokine receptor interaction and etc. were differentially enriched in the highly expressed phenotypes of PD-L1 (Fig 6C).

The landscape of immune cell infiltration of Stat3、IL-6、PD-1 and PD-L1 is shown in Fig 6D. The median mRNA expression of STAT3, IL-6, PD-1, and PD-L1 divided GC samples into high and low expression groups. The B memory cells, CD4 memory-resting T cells, CD4 memory-activated T cells, follicular helper T cells, M1 macrophages, M2 macrophages, activated mast cells were significantly different between the Stat3 groups (*P* < 0.05). The B memory cells, plasma cells, CD8 T cells, CD4 memory-activated T cells, NK resting cells, M0 macrophages, M2 macrophages, resting mast cells, activated mast cells, Neutrophils were significantly different between the IL-6 groups (*P* < 0.05). The B memory cells, Plasma cells, CD8 T cells, CD4 memory-resting T cells, CD4 memory-activated T cells, follicular helper T cells, M0 macrophages, M1 macrophages, M2 macrophages, Dendritic activated cells, resting mast cells, activated mast cells, eosinophils, neutrophils were significantly different between the PD-1 groups (*P* < 0.05). The B naive cells, B memory cells, CD8 T cells, CD4 memory-resting T cells, CD4 memory-activated T cells, follicular helper T cells, regulatory T cells (Tregs), NK resting cells, M0 macrophages, M1 macrophages, resting mast cells, neutrophils were significantly different between the PD-L1 groups (*P* < 0.05).

The mRNA expression of Stat3 was mainly positively correlated with M2 Macrophages (r = 0.14), resting mast cells (r = 0.09), etc., but negatively correlated with follicular helper T cells (r = -0.12), eosinophils (r = -0.12), etc. The expression of IL-6 mRNA was mainly positively correlated with activated mast cells (r = 0.3), neutrophils (r = 0.24), etc., and was negatively correlated with activated NK cells (r = -0.15), regulatory T cells (Tregs) (r = -0.26), etc. The expression of PD-1 mRNA was mainly positively correlated with CD4 memory-activated T cells (r = 0.38), follicular helper T cells (r = 0.25), etc., and was negatively correlated with activated mast cells (r = -0.22), CD4 memory-activated T cells (r = -0.36), etc. The mRNA expression of PD-L1 was mainly positively correlated with CD4 memory-activated T cells (r = 0.48), M1 macrophages (r = 0.29), CD8 T cells (r = 0.2), etc., and was negatively correlated with regulatory T cells (Tregs) (r = -0.28), CD4 memory-resting T cells (r = -0.3), etc. Moreover, there was a significant positive correlation between the mRNA expression levels of any combination of the two indicators of IL-6, Stat3, PD-1 and PD-L1 (p < 0.01, Fig 6E).

In addition to tumor cells, TME is mainly composed of immune cells and stromal cells which play an important role in the occurrence and development of tumors. In this study, the immune score, stromal score, and ESTIMATE score were calculated in GC patients from TCGA plus GEO combined by the ESTIMATE algorithm, and the proportion of immune cells and stromal cells in the GC microenvironment was estimated. As shown in Fig 6F, the immune score, stromal score, and ESTIMATE score in the high expression group of Stat3, IL-6, PD-1, and PD-L1 were higher than those in the low expression group (*P*<0.01).

### 3.9 TCGA dataset validates and assesses the value of the prognostic model for GC patients

**Fig 7:**
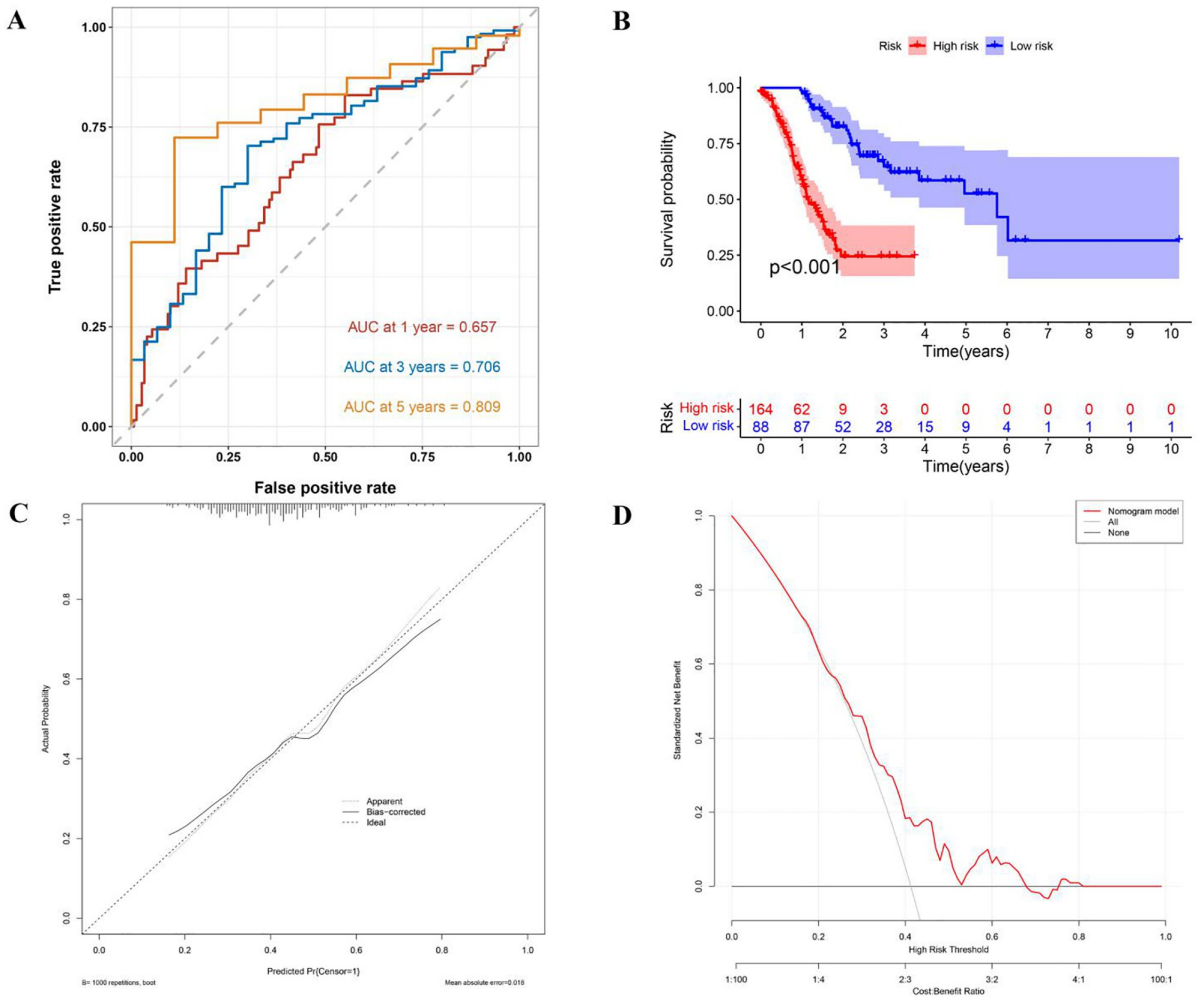
ROC curves, survival curves, calibration, and decision curve analysis for the TCGA data set. ROC curves for 3, 5 and 10 year projections of the line graph model. (B) In the validation set, the high-risk group was associated with worse survival outcomes, P< 0.001. (C) Calibration curves for the survival probability of the column line graph model. (D) The DCA curve was used to evaluate the clinical utility of the nomogram model decision strategy.

We verified the efficacy of the multifactorial model (cell differentiation+IL-6+p-Stat3+PD-1) in predicting postoperative survival status of GC in TCGA database. As shown in Fig 7, the AUCs of this GC prognostic model were 0.794, 0.896 and 0.897 at 1, 3 and 5 years, respectively, indicating that this multifactorial model had a high degree of discrimination. The mean postoperative survival time of GC patients in the low-risk group was longer than that of GC patients in the high-risk group (*P*<0.001), indicating that this model was able to accurately distinguish high-risk GC patients from low-risk GC patients. This model was externally validated using the Bootstrap method, and the results showed that the calibration curve was in good agreement with the ideal curve, suggesting that the model predicted a small difference between the patient survival rate and the true survival rate, demonstrating a high accuracy for this multifactorial model. Using DCA to assess the clinical application value of this model, the net benefit of the model was relatively high and the results showed a high clinical value for this multifactorial model.

## 4. Discussion

Gastric cancer is one of the major cancer burdens worldwide. Many influential factors play a role in the development and progression of GC. Surgery is the major treatment for gastric cancer but its efficacy depends on the advancement of the disease. For example, early diagnosed GC has a 5-year survival rate of >90% after surgery but advanced GC often has a poor prognosis [13].

Population screening is effective in early diagnosis of GC, however, the cost burden, screening facilities/professionals and individuals’ awareness are realistic barriers for such screening in developing and less developed countries. Before more advanced and/or cost-effective screening techniques for GC are available, how to evaluate potential survivorship and quality of life after surgery among advanced GC patients would be one realistic task for medical professionals to consider at the present. This study has tested the hypothesis that some signaling pathways and their molecules associated with cancers may be candidates to serve as prognostic markers in predicting the survival time in postoperative GC patients.

IL-6/Stat3 signaling pathway promotes the proliferation, invasion and lymphangiogenesis of gastric cancer cells by stimulating JAK-STAT3-VEGF-C signaling pathway [14]. This study has showed that IL-6 was expressed at higher levels in GC tissues (S2 Table). According to previous studies [15, 16], IL-6 levels increase substantially in the serum and cancer tissues of GC patients. Continuous follow-up of GC patients who underwent surgery reveals that IL-6 concentrations are higher in the serum of GC patients before surgery but decrease after surgery [17]. Furthermore, serum IL-6 levels are significantly higher in relapsed patients while decreased in patients with no relapses.

On the other hand, Stat3 is a crucial mediator of carcinogenesis through tumor-associated immunosuppression, implying a key role in tumor cell proliferation, invasion, metastasis, and immune escape [18–20]. As phosphorylated Stat3 (p-Stat3) is the active form of the molecule, we therefore have tested p-Stat3 in this study. As shown in Table 1, the levels of p-Stat3 are higher in GC tissues than in ANT tissues, in keeping with the increased expression of p-Stat3 in most malignant tumors tested [21–24]. The above observations suggest that molecules of the IL-6/Stat3 pathway play important roles in cancers and in this context, these molecules may be valid biomarkers for the diagnosis as well as the prognosis for GC patients.

PD-1 is expressed in various immune cells (such as myeloid cells, T cells, B cells) [25] and functions as an immune checkpoint molecule that binds to its ligand to inhibit T cell activation acting as an immune "brake" [26]. PD-1 inhibits T cell activation and regulates the effector properties of CD8+ T cells. In the tumor microenvironment, this inhibitory pathway plays a different role than in the normal microenvironment as tumor cells take advantage of this pathway to express PD-L1 (and PD-L2) on their surface. Obviously, PD-1/PD-L1 pathway also plays important roles in the development and progression of cancer through immune response and immune regulation [27–29].

In this study, the expressed levels of IL-6 are correlated with distant metastasis and clinical stage (S3 Table), suggesting a possible involvement of IL-6 in local and distant metastasis in GC. The activated levels of p-Stat3 have correlated with cell differentiation, the depth of infiltration, lymph node metastasis, TNM staging, suggesting a broad involvement in the development and progression of GC.

On the other hand, we have found that PD-1 expression levels are not correlated with patients’ gender, age, cell differentiation, depth of infiltration, lymph node metastasis, distant metastasis and TNM staging (S5 Table), which is consistent with the findings by others [30]. However, PD-L1 expression is correlated with cell differentiation, depth of infiltration, and TNM staging (S5 Table). These correlation differences between PD-1 and PD-L1 may be due to the presence of other signaling molecules that synergistically participate in the immunosuppressive response of T cells with the PD-1/PD-L1 pathway [31]. In this study, the PD-L1 expression positive group has a better survival rate and PD-L1 is, therefore, an important factor affecting the survival prognosis in GC patients.

We have observed that IL-6, Stat3, PD-1 and PD-L1 are associated not only within the signaling pathways but also with multiple immune cells and tumor microenvironment (Fig 6). For example, Activated Stat3 enters the nucleus of cells and binds to the promoter of PD-L1 gene and induces the expression of PD-L1 on the surface of tumor cells. PD-L1 in tumor tissues, in turn, induces effector T cell death and inhibits CD8+ T cell activation after binding to PD-1, thus allowing tumor cells to escape from the body’s immune surveillance and killing [32–35].

As shown in this study, highly expressed IL-6, Stat3, PD-1 and PD-L1 mRNA are all significantly enriched in immune-related signaling pathways, suggesting that Stat3 signaling may play an important role in interfering immune response (anti-immune response) in tumor microenvironment whereby promoting the growth and progression of tumor. In tumor cells themselves, over-activated Stat3 reduces the expression of immunostimulatory factors by which exert profound immune effects [36]. Stat3 signaling interacts with other signaling pathways to confer stability for tumor progression [37]. In addition, studies have also revealed that over-activated Stat3 in tumor cells can inhibit the maturation of dendritic cells and innate immunity, thereby reducing the anti-tumor effector function of CD8+T cells [38, 39]. Furthermore, in adaptive immune subsets, increased Stat3 activity inhibits the accumulation of effector T cells by which inhibit their anti-tumor effects [40, 41]. This is consistent with our findings that Stat3 activity is negatively correlated with regulatory T cells in gastric cancer.

On the other hand, our results here have shown that PD-L1 mRNA expression is negatively correlated with CD4 T cells and NK cells. PD-L1 and PD-1 signaling pathway can inhibit the anti-tumor immune response of T cells and promote immune escape of tumor cells [42]. Reports have shown that tumors with high PD-L1 expression have a significantly reduced proportion of infiltrating CD4+T lymphocytes, natural killer cells, and monocytes [43]. These observations may explain a phenomenon that an accelerated tumor growth is often seen after up-regulated expression of PD-L1 in tumor cells [44].

Like many other cancers, gastric cancer is polygenic which predicts a multi-staged pathogenesis in the development and progression of the disease [45]. Therefore, it is conceivable that the ability of a single gene or its product is limited in the diagnosis and/or prognosis of GC. We favor the hypothesis that multiple biomarkers should be more informative when investigating prognostic risk factors for GC patients.

Kaplan-Meier survival analysis compares groupings within one variable for its power (ability) to discriminate differences in survival, but cannot make comparisons between or among different survival variables because they are not quantified. We have first used ROC curve and AUC (area under the ROC curve) to quantify the power of a survival predictor in GC patients, by which compares the potentials of different survival predictors [46]. The larger the AUC is, the more powerful the risk factor can be in predicting prognosis. As shown in Fig 4, in terms of predicting survival in postoperative GC patients, AUCs are 0.663 for IL-6, 0.601 for p-Stat3, and 0.593 for PD-L1, respectively, indicating that IL-6, p-Stat3 and PD-L1 have power (ability) in predicting survival prognosis for GC patients. TNM staging is a well-established standard in predicting prognosis for cancer patients and, of cause in this study, TNM shows the largest AUC (Table 2), indicating that TNM is the most powerful predictor in terms of single predictor comparisons in GC patients.

As mentioned above, we favor the hypothesis that multiple survival predictors should be more informative in predicting survival prognosis. We have then tested this hypothesis in this study. As shown in S5 Table, the combinations of multiple predictors indeed show higher AUC in predicting survival of GC patients. To our surprise, however, the combination of 4 predictors, "cell differentiation+IL-6+p-Stat3+PD-1", has the largest AUC (0.782) which is even larger than the AUC of the standard TNM staging (0.742) (S5 Table). These observations have demonstrated a high predicting power using multiple predictors in combination and confirmed the above hypothesis. Furthermore, the results of ROC curve, calibration, DCA and Kaplan-Meier (KM) survival curves in TCGA dataset confirmed that the nomogram model could accurately predict the prognosis of patients with gastric cancer.

Our previous studies have demonstrated that nomogram analysis using bio-indicators to predict OS among GC patients has advantages over TNM staging. Classic TNM staging is only possible in predicting survival after surgery. We have previously introduced a nomogram based on patients’ basic clinical features and preoperative bio-indicators. The nomogram can predict the 5-year OS among GC patients with a reliable performance (AUC of 0.78) [38]. In this study, we have constructed a nomogram model that includes grading as well as immunohistochemical indicators and found that nomograms show more accurate predicting power than that of TNM stage alone (Figs 4 and 5), in keeping with our previous observations [47].

Multi-omics integrates information at different levels, builds a gene regulatory network, and deeply understands the regulation and causal relationship between various molecules, so as to gain a deeper understanding of the molecular mechanism(s) and genetic basis of complex traits in the progression of gastric cancer. Studies have shown that multi-omics-based liver cancer models provide better predictive indicators than single-omics-based models [48, 49], in agreement with our present observations in a multifactorial prognostic model for postoperative GC patients.

In summary, we have successfully constructed a nomogram multi-predictor model for survival prognosis among postoperative GC patients in our own database, which has been validated in the external TCGA database. The observations here have demonstrated that this nomogram model is capable of predicting 1-year, 3-year and 5-year survival rates, respectively, among postoperative GC patients. Because these bio-predictors, IL-6, p-Stat3, PD-1 and PD-L1, can be examined in diagnostic biopsies before surgery, this multi-predictor model may have clinical usefulness in helping clinicians’ decision-makings, including but not limited to, treatment options and follow-up plans before surgery as well as after surgery.

## Supporting information

S1 Table Patients’ clinicopathologic characteristics. (DOCX)

S2 Table Comparisons of IHC-based indicators of IL-6, p-Stat3, PD-1 and PD-L1 between cancer and adjacent tissues. (DOCX)

S3 Table The relationship between the expression of IL-6 and p-Stat3 in gastric cancer tissues and the clinicopathological characteristics of patients. (DOCX)

S4 Table The relationship between the expression of PD-1 and PD-L1 in the tissues of patients with gastric cancer and the clinicopathological characteristics of patients. (DOCX)

S5 Table AUCs (area under the curves) for various bio-indicator combinations in gastric cancer patients. (DOCX)

S1 Fig Hosmer-Lemeshow goodness-of-fit test curve of multi-indicator joint prediction model. (PDF)
