# Supplementary material for: A cohort study using IL-6/Stat3 activity and PD-1/PD-L1 expression to predict five-year survival for patients after gastric cancer resection
Source: PLoS One. 2022 Dec 1;17(12):e0277908. doi: 10.1371/journal.pone.0277908 (PMC9714712; doi:10.1371/journal.pone.0277908)
Supplement: S1 Table — (DOCX) [file pone.0277908.s001.docx]

| **S1 Table Patients’ clinicopathologic characteristics** | | |
| --- | --- | --- |
| Clinicopathologic Characteristic | Mean or No. of patients（n=205) | SD or % |
| Gender |  |  |
| male | 154 | 75.13% |
| female | 51 | 24.87% |
| Age | 62.17 | 10.73 |
| ＜60 | 76 | 37.07% |
| ≥60 | 129 | 62.93% |
| Differentiation |  |  |
| high | 9 | 4.41% |
| moderate | 56 | 27.45% |
| low | 139 | 68.14% |
| T |  |  |
| T₁ | 26 | 12.75% |
| T₂ | 41 | 20.09% |
| T₃ | 103 | 50.49% |
| T_4_ | 34 | 16.67% |
| N |  |  |
| no | 78 | 38.24% |
| yes | 126 | 61.76% |
| M |  |  |
| no | 186 | 91.18% |
| yes | 18 | 8.82% |
| Clinical stages |  |  |
| Ⅰ | 45 | 22.17% |
| Ⅱ | 69 | 33.99% |
| Ⅲ | 71 | 34.98% |
| Ⅳ | 18 | 8.87% |
| Follow-up, months | 59.47 | 39.698 |
| Abbreviations: SD, standard deviation; T, depth of invasion; N, ymph node metastasis; M,distant metastasis. | | |
