# Supplementary material for: A cohort study using IL-6/Stat3 activity and PD-1/PD-L1 expression to predict five-year survival for patients after gastric cancer resection
Source: PLoS One. 2022 Dec 1;17(12):e0277908. doi: 10.1371/journal.pone.0277908 (PMC9714712; doi:10.1371/journal.pone.0277908)
Supplement: S2 Table — (DOCX) [file pone.0277908.s002.docx]

**S2 Table Comparisons of IHC-based indicators of IL-6 ,p-Stat3 ,PD-1 and PD-L1 between cancer and adjacent tissues**

| Grouping | n | - | 1+ | 2+ | 3+ | Positive rate | χ² | *P* |
| --- | --- | --- | --- | --- | --- | --- | --- | --- |
| IL-6 |  |  |  |  |  |  |  |  |
| Gastric cancer | 179 | 34(18.99%) | 63(35.20%) | 49(27.37%) | 33(18.44%) | 81.01% | 68.888 | ＜0.001 |
| adjacent | 180 | 85(47.22%) | 77(42.78%) | 17(9.44%) | 1(0.56%) | 52.78% |  |  |
| p-Stat3 |  |  |  |  |  |  |  |  |
| Gastric cancer | 187 | 0(0.00%) | 19(10.16%) | 29(15.51%) | 139(74.33%) | 100.00% | 153.001 | ＜0.001 |
| adjacent | 167 | 11(6.59%) | 57(34.13%) | 83(49.70%) | 16(9.58%) | 93.41% |  |  |
| PD-1 |  |  |  | | |  |  |  |
| Gastric cancer | 169 | 70(41.42%) | 99(58.58%) | | | 99(58.58%) | 11.271 | ＜0.001 |
| adjacent | 162 | 97(59.88%) | 65(40.12%) | | | 65(40.12%) |  |  |
| PD-L1 |  |  |  | | |  |  |  |
| Gastric cancer | 178 | 110(61.80%) | 68(38.20%) | | | 68(38.20%) | 5.065 | 0.025 |
| adjacent | 171 | 125(73.10%) | 46(26.90%) | | | 46(26.90%) |  |  |
| Note: p <0.05 indicates significant statistical differences. | | | | | | | | |
