# Supplementary material for: A cohort study using IL-6/Stat3 activity and PD-1/PD-L1 expression to predict five-year survival for patients after gastric cancer resection
Source: PLoS One. 2022 Dec 1;17(12):e0277908. doi: 10.1371/journal.pone.0277908 (PMC9714712; doi:10.1371/journal.pone.0277908)
Supplement: S3 Table — (DOCX) [file pone.0277908.s003.docx]

**S3 Table The relationship between the expression of IL-6 and p-Stat3 in gastric cancer tissues and the clinicopathological characteristics of patients**

|  | n | IL-6 | | | *P* values | n | p-Stat3 | | | *P* values |
| --- | --- | --- | --- | --- | --- | --- | --- | --- | --- | --- |
|  |  | -/1+(%) | 2+(%) | 3+(%) |  |  | -/1+(%) | 2+(%) | 3+(%) |  |
| Gender |  |  |  |  |  |  |  |  |  |  |
| male | 134 | 76(56.72%) | 35(26.12%) | 23(17.16%) | 0.498 | 139 | 14(10.07%) | 20(14.39%) | 105(75.54%) | 0.761 |
| female | 45 | 21(46.67%) | 14(31.11%) | 10(22.22%) |  | 48 | 5(10.42%) | 9(18.75%) | 34(70.83%) |  |
| Age |  |  |  |  |  |  |  |  |  |  |
| ＜60 | 64 | 38(59.38%) | 16(25.00%) | 10(15.62%) | 0.571 | 71 | 7(9.86%) | 13(18.31%) | 51(71.83%) | 0.711 |
| ≥60 | 115 | 59(51.30%) | 33(28.70%) | 23(20.00%) |  | 116 | 12(10.34%) | 16(13.80%) | 88(75.86%) |  |
| Differentiation |  |  |  |  |  |  |  |  |  |  |
| high | 7 | 5(71.42%) | 1(14.29%) | 1(14.29%) | 0.159 | 7 | 2(28.57%) | 1(14.29%) | 4(57.14%) | 0.052 |
| moderate | 50 | 30(60.00%) | 15(30.00%) | 5(10.00%) |  | 50 | 8(16.00%) | 9(18.00%) | 33(66.00%) |  |
| low | 121 | 61(50.41%) | 33(27.27%) | 27(22.32%) |  | 129 | 9(6.98%) | 19(14.73%) | 101(78.29%) |  |
| T |  |  |  |  |  |  |  |  |  |  |
| T₁ | 16 | 10(62.50%) | 4(25%) | 2(12.50%) | 0.720 | 20 | 0(0.00%) | 3(15.00%) | 17(85.00%) | **0.044** |
| T₂ | 34 | 19(55.88%) | 9(26.47%) | 6(17.65%) |  | 36 | 10(27.78%) | 9(25.00%) | 17(47.22%) |  |
| T₃ | 100 | 53(53.00%) | 25(25.00%) | 22(22.00%) |  | 102 | 8(7.84%) | 15(14.71%) | 79(77.45%) |  |
| T_4_ | 27 | 14(51.85%) | 10(37.04%) | 3(11.11%) |  | 27 | 1(3.70%) | 2(7.41%) | 24(88.89%) |  |
| N |  |  |  |  |  |  |  |  |  |  |
| no | 73 | 46(63.01%) | 18(24.66%) | 9(12.33%) | 0.097 | 72 | 13(18.06%) | 18(25.00%) | 41(56.94%) | **＜0.001** |
| yes | 104 | 50(48.08%) | 30(28.84%) | 24(23.08%) |  | 113 | 6(5.31%) | 11(9.73%) | 96(84.96%) |  |
| M |  |  |  |  |  |  |  |  |  |  |
| no | 162 | 92(56.79%) | 44(27.16%) | 26(16.05%) | **0.010** | 167 | 19(11.37%) | 27(16.17%) | 121(72.46%) | 0.236 |
| yes | 15 | 4(26.67%) | 4(26.67%) | 7(46.66%) |  | 18 | 0(0.00%) | 2(11.11%) | 16(88.89%) |  |
| Clinical stages |  |  |  |  |  |  |  |  |  |  |
| Ⅰ | 36 | 22(61.11%) | 11(30.56%) | 3(8.33%) | **0.005** | 40 | 7(17.50%) | 9(22.50%) | 24(60.00%) | **0.001** |
| Ⅱ | 61 | 40(65.58%) | 12(19.67%) | 9(14.75%) |  | 60 | 10(16.67%) | 11(18.33%) | 39(65.00%) |  |
| Ⅲ | 65 | 30(46.15%) | 21(32.31%) | 14(21.54%) |  | 67 | 2(2.99%) | 7(10.44%) | 58(86.57%) |  |
| Ⅳ | 15 | 4(26.67%) | 4(26.67%) | 7(46.66%) |  | 18 | 0(0.00%) | 2(11.11%) | 16(88.89%) |  |
| Note: *P* <0.05 indicates significant statistical differences. | | | | | | | | | | |
