# Supplementary material for: A cohort study using IL-6/Stat3 activity and PD-1/PD-L1 expression to predict five-year survival for patients after gastric cancer resection
Source: PLoS One. 2022 Dec 1;17(12):e0277908. doi: 10.1371/journal.pone.0277908 (PMC9714712; doi:10.1371/journal.pone.0277908)
Supplement: S4 Table — (DOCX) [file pone.0277908.s004.docx]

**S4 Table The relationship between the expression of PD-1 and PD-L1 in the tissues of patients with gastric cancer and the clinicopathological characteristics of patients**

|  | n | PD-1 | | *P* values | n | PD-L1 | | *P* values |
| --- | --- | --- | --- | --- | --- | --- | --- | --- |
|  |  | -(%) | +(%) |  |  | -(%) | +(%) |  |
| Gender |  |  |  |  |  |  |  |  |
| male | 129 | 55(42.64%) | 74(57.36%) | 0.566 | 134 | 79(58.96%) | 55(41.04%) | 0.174 |
| female | 40 | 15(37.50%) | 25(62.50%) |  | 44 | 31(70.45%) | 13(29.55%) |  |
| Age |  |  |  |  |  |  |  |  |
| ＜60 | 60 | 26(43.33%) | 34(56.67%) | 0.709 | 63 | 41(65.08%) | 22(34.92%) | 0.506 |
| ≥60 | 109 | 44(40.37%) | 65(59.63%) |  | 115 | 69(60.00%) | 46(40.00%) |  |
| Differentiation |  |  |  |  |  |  |  |  |
| high | 7 | 4(57.14%) | 3(42.86%) | 0.285 | 6 | 6(100.00%) | 0(0.00%) | **0.013** |
| moderate | 49 | 22(44.90%) | 27(55.10%) |  | 46 | 33(71.74%) | 13(28.26%) |  |
| low | 112 | 43(38.39%) | 69(61.61%) |  | 125 | 70(56.00%) | 55(44.00%) |  |
| T |  |  |  |  |  |  |  |  |
| T₁ | 16 | 7(43.75%) | 9(56.25%) | 0.237 | 19 | 15(78.95%) | 4(21.05%) | **0.008** |
| T₂ | 33 | 18(54.55%) | 15(45.45%) |  | 31 | 27(87.10%) | 4(12.90%) |  |
| T₃ | 93 | 34(36.56%) | 59(63.44%) |  | 99 | 49(49.49%) | 50(50.51%) |  |
| T_4_ | 25 | 10(40.00%) | 15(60.00%) |  | 27 | 17(62.96%) | 10(37.04%) |  |
| N |  |  |  |  |  |  |  |  |
| no | 67 | 22(32.84%) | 45(67.16%) | 0.069 | 69 | 48(69.57%) | 21(30.43%) | 0.074 |
| yes | 100 | 47(47.00%) | 53(53.00%) |  | 107 | 60(56.07%) | 47(43.93%) |  |
| M |  |  |  |  |  |  |  |  |
| no | 151 | 61(40.40%) | 90(59.60%) | 0.460 | 160 | 101(63.12%) | 59(36.88%) | 0.130 |
| yes | 16 | 8(50.00%) | 8(50.00%) |  | 16 | 7(43.75%) | 9(56.25%) |  |
| Clinical stages |  |  |  |  |  |  |  |  |
| Ⅰ | 35 | 15(42.86%) | 20(57.14%) | 0.986 | 34 | 28(82.35%) | 6(17.65%) | **＜0.001** |
| Ⅱ | 56 | 23(41.07%) | 33(58.93%) |  | 61 | 42(68.85%) | 19(31.15%) |  |
| Ⅲ | 60 | 23(38.33%) | 37(61.67%) |  | 65 | 31(47.69%) | 34(52.31%) |  |
| Ⅳ | 16 | 8(50.00%) | 8(50.00%) |  | 16 | 7(43.75%) | 9(56.25%) |  |
| Note:*P* <0.05 indicates significant statistical differences. | | | | | | | | |
