# Supplementary material for: A cohort study using IL-6/Stat3 activity and PD-1/PD-L1 expression to predict five-year survival for patients after gastric cancer resection
Source: PLoS One. 2022 Dec 1;17(12):e0277908. doi: 10.1371/journal.pone.0277908 (PMC9714712; doi:10.1371/journal.pone.0277908)
Supplement: S5 Table — (DOCX) [file pone.0277908.s005.docx]

**S5 Table AUCs (area under the curves) for various bio-indicator combinations in gastric cancer patients**

| Index | AUC | 95% CI | *P* values | Sensitivity | Specificity | Youden index | Cut-offvalues |
| --- | --- | --- | --- | --- | --- | --- | --- |
| 1.Differentiation+IL-6+p-Stat3+PD-1+PD-L1 | 0.777 | 0.701-0.854 | ＜0.001 | 89.47% | 53.12% | 0.426 | 0.378 |
| 2.Differentiation+IL-6+p-Stat3+PD-1 | 0.782 | 0.709-0.856 | ＜0.001 | 81.25% | 61.97% | 0.432 | 0.395 |
| 3.IL-6+p-Stat3+PD-1 | 0.770 | 0.695-0.845 | ＜0.001 | 80.00% | 62.50% | 0.425 | 0.474 |
| 4.IL-6+p-Stat3 | 0.740 | 0.665-0.814 | ＜0.001 | 54.95% | 77.63% | 0.326 | 0.592 |
| 5.TNM | 0.742 | 0.675-0.809 | ＜0.001 | 62.39% | 77.66% | 0.401 | 2.500 |
| Note: *P* <0.05 indicates significant statistical differences. | | | | | | | |
