# Supplementary material for: A cohort study using IL-6/Stat3 activity and PD-1/PD-L1 expression to predict five-year survival for patients after gastric cancer resection
Source: PLoS One. 2022 Dec 1;17(12):e0277908. doi: 10.1371/journal.pone.0277908 (PMC9714712; doi:10.1371/journal.pone.0277908)
Supplement: S1 Fig — (PDF) [file pone.0277908.s006.pdf]

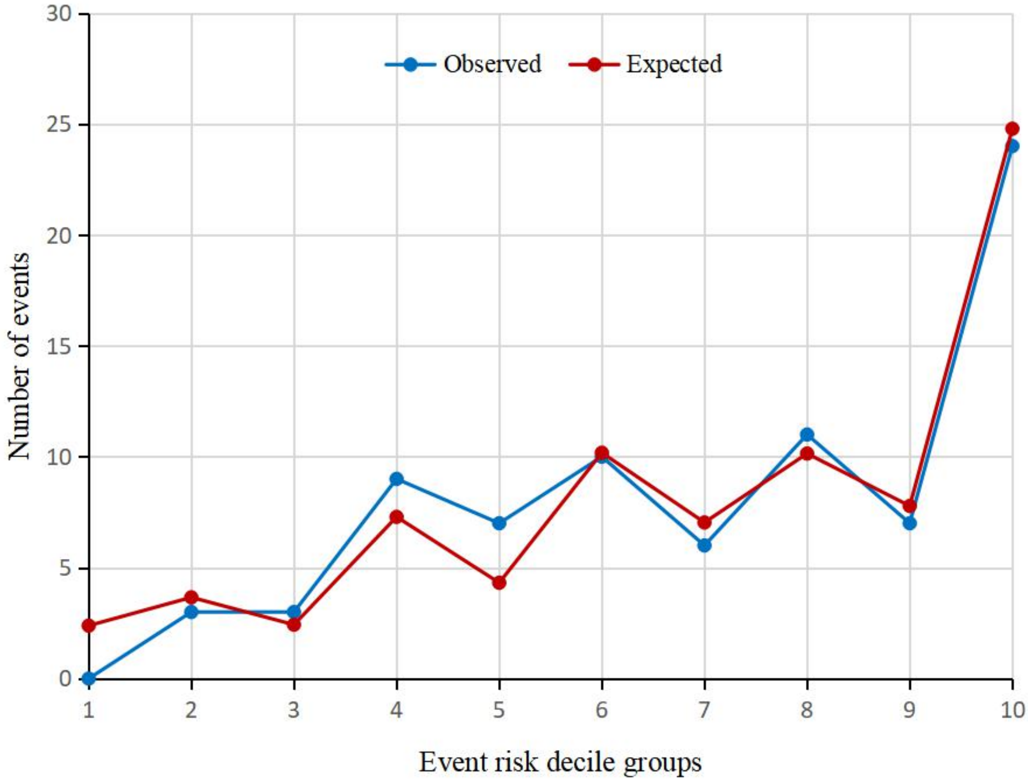

S1 Fig Hosmer-Lemeshow goodness-of-fit test curve of multi-indicator joint prediction model

The Hosmer-Lemeshow goodness-of-fit test was used to divide the sample data into 10 groups based on the predicted probability from smallest to largest, and the calibration curve of this combined model was plotted using coordinate points indicating the magnitude of the actual observed values versus the values derived from the prediction model. The calibration level is higher when the curve of predicted values is closer to the curve of actual observed values.
